# Supplementary material for: Indels allow antiviral proteins to evolve functional novelty inaccessible by missense mutations
Source: bioRxiv. 2024 May 10:2024.05.07.592993. Preprint. [Version 1] doi: 10.1101/2024.05.07.592993 (PMC11100679; doi:10.1101/2024.05.07.592993)
Supplement: Supplement 6 [file NIHPP2024.05.07.592993v1-supplement-6.pdf]

bioRxiv preprint doi: https://doi.org/10.1101/2024.05.07.592993; this version posted May 10, 2024. The copyright holder for this preprint (which was not certified by peer review) is the author/funder, who has granted bioRxiv a license to display the preprint in perpetuity. It is made available under aCC-BY-NC 4.0 International license.

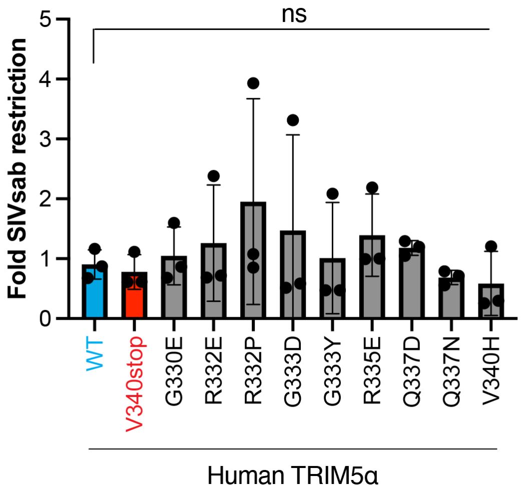

Figure S1: Validation of human TRIM5α deep mutational scanning results. Single missense variants of human TRIM5α (which were not enriched in the gain-of-SIVsab restriction screen in Fig. 1C) were stably expressed in CRFK cells and challenged with SIVsab; fold restriction was calculated as an increase in infectious dose (ID_5) compared to cells expressing empty vector. Error bars, SD; ns, not significant; Kruskal-Wallis test with Dunn's multiple comparison (vs. WT) correction.

bioRxiv preprint doi: https://doi.org/10.1101/2024.05.07.592993; this version posted May 10, 2024. The copyright holder for this preprint (which was not certified by peer review) is the author/funder, who has granted bioRxiv a license to display the preprint in perpetuity. It is made available under aCC-BY-NC 4.0 International license.

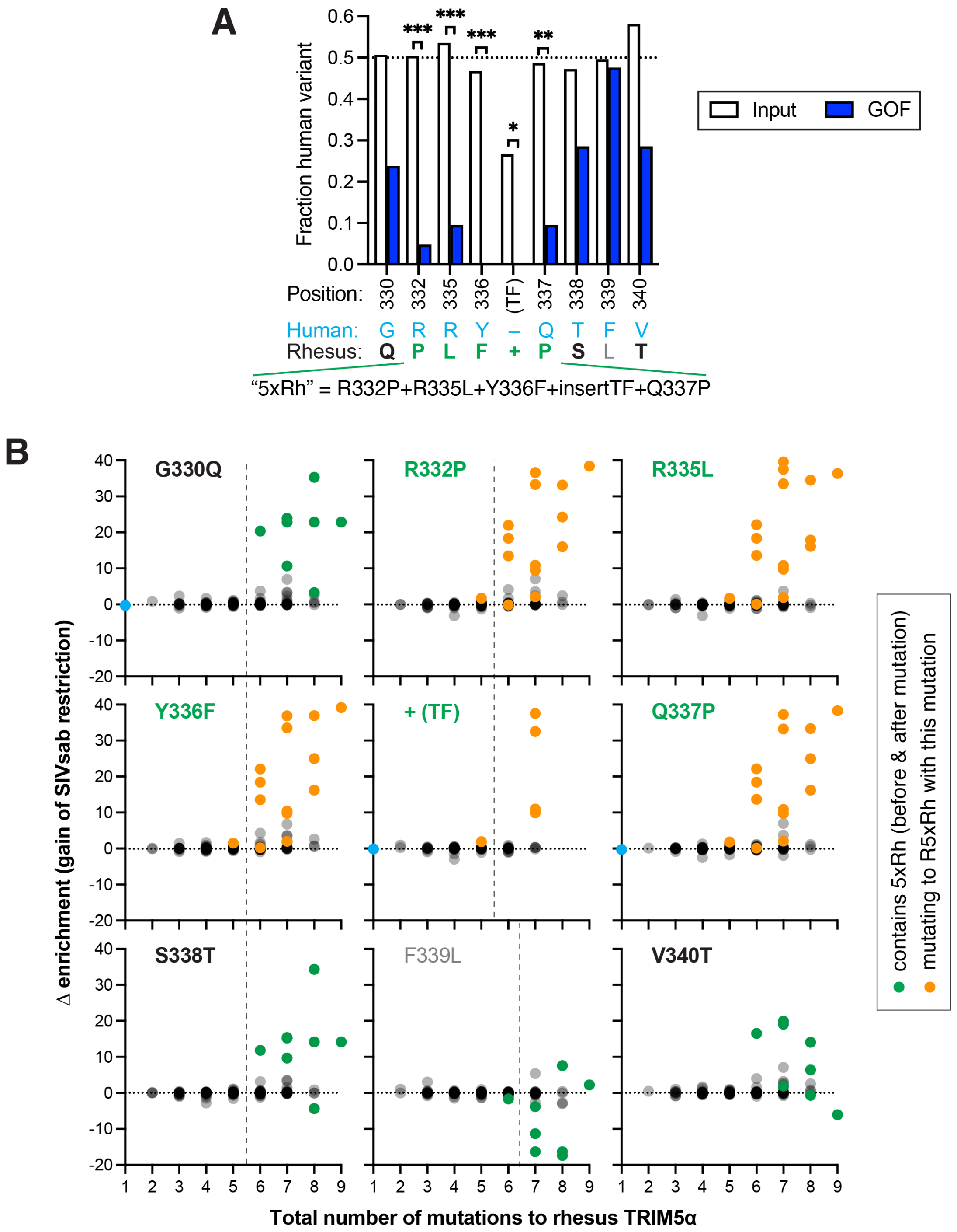

Figure S2: No rhesus-like mutation confers SIVsab restriction onto human TRIM5α in the absence of at least 5 other mutations. (A) The fraction of variants encoding the human sequence at each position for the input library (all variants detected in unsorted cells) and gain-of-function (GOF) variants (defined as outscoring all frameshift variants [see Materials and Methods for frameshift origin] in both replicates). The 5 statistically required rhesus mutations (5xRh) are highlighted in green; other beneficial rhesus mutations (positions that display a modest preference against the human variant) are bolded in black. *$p < 0.05$; **$p < 0.01$; ***$p < 0.001$; Chi-square test. **(B)** The change in enrichment (in SIVsab restrictor pool) as human TRIM5α variants acquire a required (green), beneficial (black), or other (gray) rhesus TRIM5α mutation was calculated by subtracting the enrichment score for each variant lacking the mutation from the matched variant that acquired the mutation but was otherwise identical in sequence. Variants are plotted by the total number of rhesus TRIM5α mutations (after acquiring the indicated mutation). Matched pairs are colored by WT starting variant (cyan), variant matched pairs that both contain the 5xRh required mutations (green), and variants that acquire all 5xRh mutations after they acquire the indicated mutation (orange). The dashed line at y = 0 indicates neutral mutations with no effect on SIVsab restriction. The dashed line at x = 5.5 highlights the requirement for 5 pre-existing mutations (6 total upon acquisition of the indicated mutation) to improve SIVsab restriction (Δ enrichment > 0) in all backgrounds. The F339L mutation does not contribute to restriction, and a 7th mutation is required for F339L variants.

bioRxiv preprint doi: https://doi.org/10.1101/2024.05.07.592993; this version posted May 10, 2024. The copyright holder for this preprint (which was not certified by peer review) is the author/funder, who has granted bioRxiv a license to display the preprint in perpetuity. It is made available under aCC-BY-NC 4.0 International license.

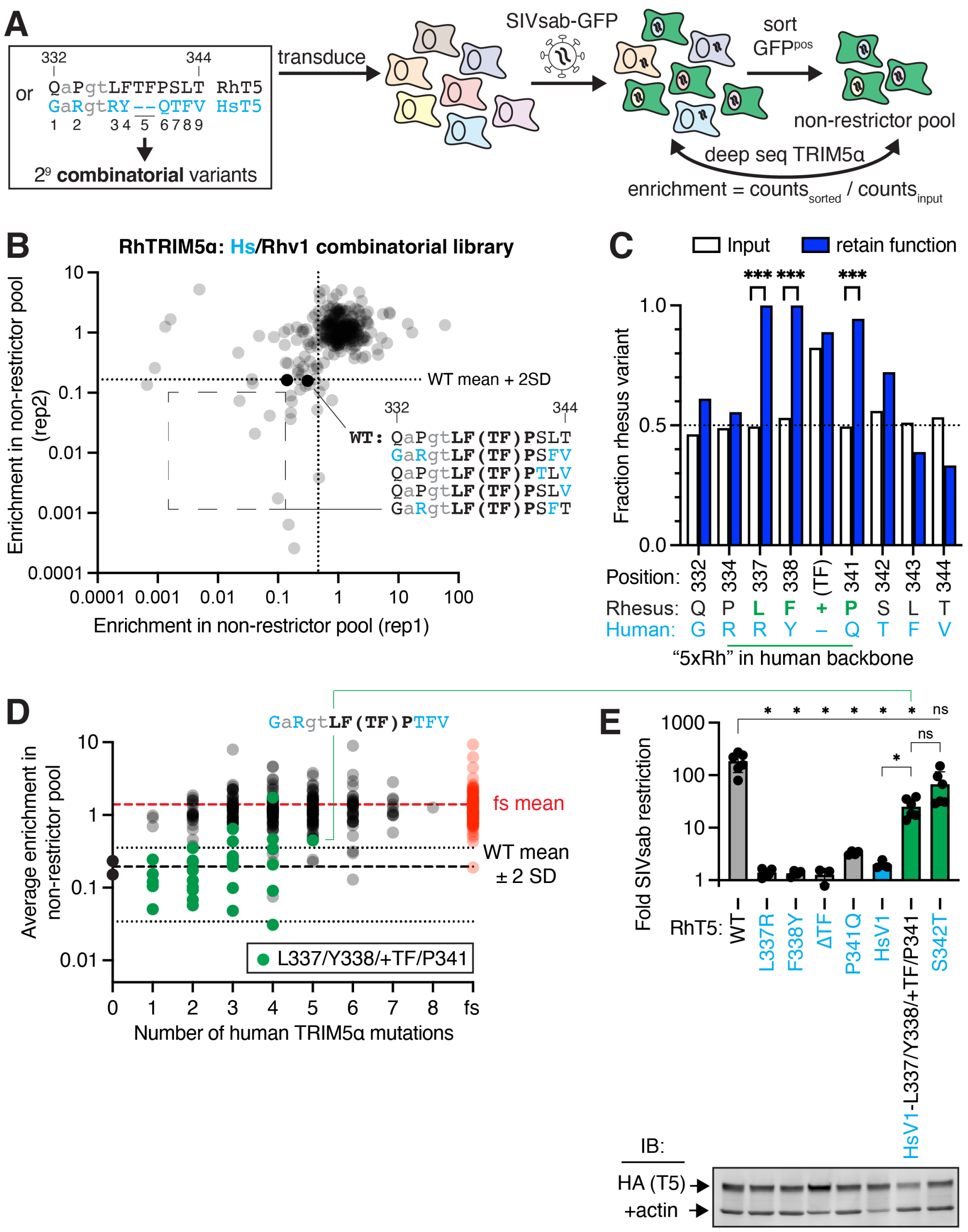

Figure S3: Rhesus TRIM5α shares the requirement for rhesus-like sequence at 4 positions (337-341) of the 5 required by human TRIM5α. (A) A rhesus TRIM5α library combinatorially sampling the human or rhesus variant at each v1 position was expressed in CRFK cells, challenged with SIVsab-GFP, and sorted for GFP[pos] cells to identify loss-of-function variants. **(B)** Most combinatorial variants (80%) are significantly enriched (> [WT (black) mean + 2 SD] in 2 biological replicates), decreasing the sensitivity of sequence preference analysis; subsequent analysis therefore focuses on the non-enriched, retention-of-function variants. The sequence of best-performing variants (dashed box) is colored by human (cyan) or rhesus (black) variant at each site. **(C)** The fraction of variants encoding the rhesus TRIM5α sequence at each position for the input library (all variants detected in unsorted cells) and retention-of-function pools (defined as < [WT mean + 2 SD] in both replicates) highlights a requirement for the rhesus TRIM5α variant at 4 central v1 loop positions; these are a subset of the 5 rhesus-like mutations strictly required by human TRIM5α. Variants lacking the (TF) insertion were under-represented in the input library, reducing statistical power, but the requirement for the insertion was confirmed by mutagenesis in (E). **(D)** Average enrichment of variants as a function of number of human v1 mutations. Variants retaining rhesus sequence at positions 337-341 (green) behave similarly to WT (black, 0 mutations), whereas other variants do not. **(E)** Rhesus TRIM5α variants were individually expressed in CRFK cells and challenged with SIVsab to confirm screen results. Rhesus variants at positions 337-341 are all individually required, and these 4 positions are sufficient to retain most SIVsab restriction in an otherwise humanized v1 loop, to levels similar to an S342T slightly impaired variant. Stable expression of all TRIM5α-HA constructs in CRFK was confirmed by immunoblot against HA. Error bars, SD. ns, not significant; *$p < 0.05$; ***$p < 0.001$; (C) Chi-square test; (E) Welch ANOVA with Dunnett's T3 multiple comparisons correction.

bioRxiv preprint doi: https://doi.org/10.1101/2024.05.07.592993; this version posted May 10, 2024. The copyright holder for this preprint (which was not certified by peer review) is the author/funder, who has granted bioRxiv a license to display the preprint in perpetuity. It is made available under aCC-BY-NC 4.0 International license.

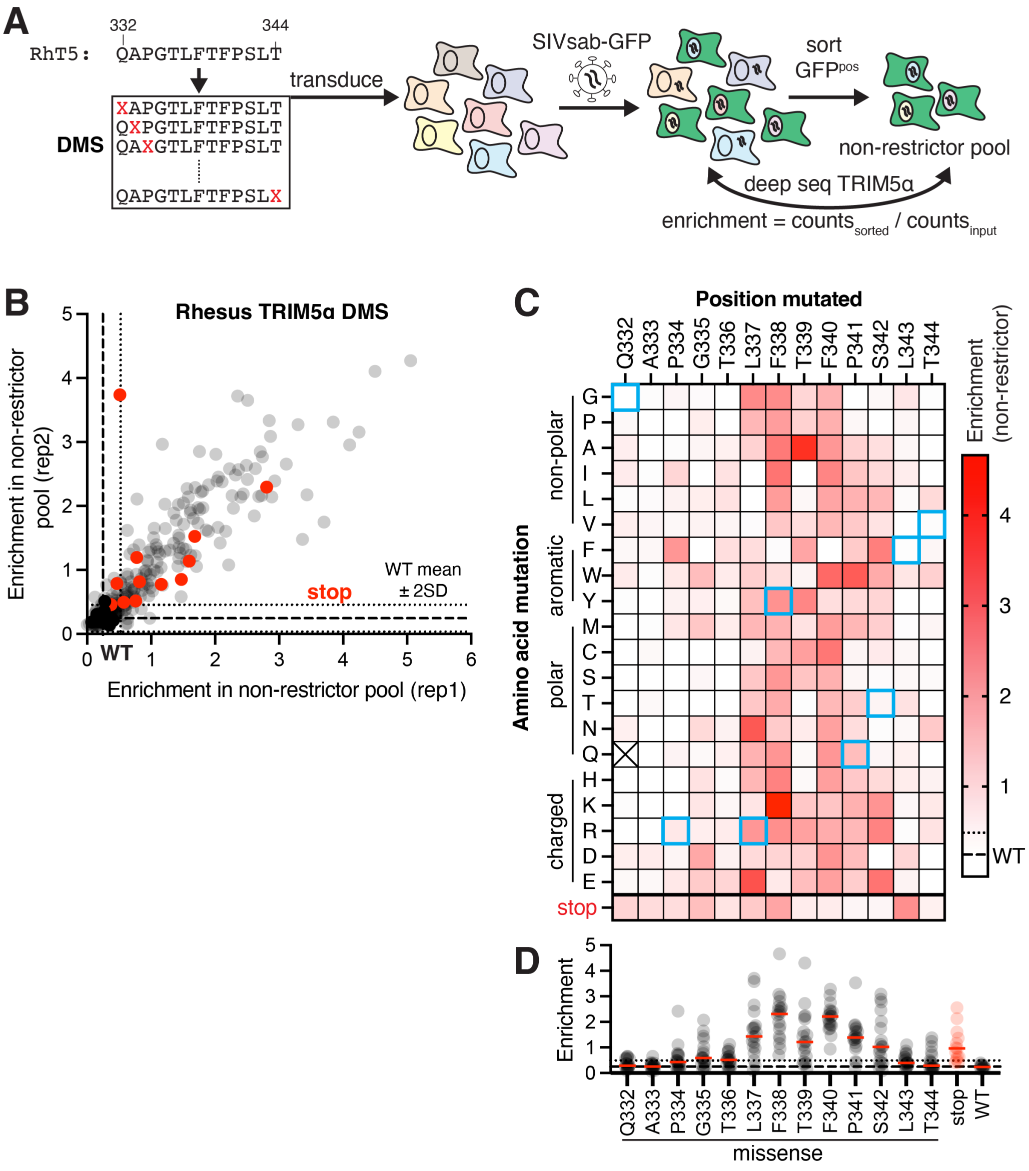

Figure S4: Most missense mutations in central v1 loop positions of rhesus TRIM5α strongly disrupt SIVsab restriction. (A) CRFK cells expressing a DMS library of rhesus TRIM5α were infected with SIVsab-GFP, and GFP^{pos} cells were sorted to identify loss-of-function variants. **(B)** Both missense (gray) and truncated variants (red) are enriched in the non-restrictor pool compared to WT (black; dashed and dotted lines: WT mean +/- 2 SD). **(C)** Average enrichment in the non-restrictor pool for each missense variant, represented as a heat map. Rhesus TRIM5α variants with a single human TRIM5α missense mutation are boxed in cyan. **(D)** The median enrichment score for missense mutations at each position (red dash) highlights mutational intolerance at positions 337-342. Error bars, SD. ns, not significant; $^{**}p < 0.01$; one-way ANOVA with Dunnett's T3 multiple comparisons test vs. WT.

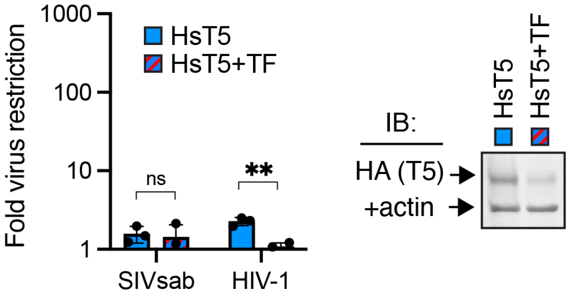

Figure S5: A rhesus-like insertion is insufficient to confer human TRIM5α with antiviral function. Human TRIM5α containing the rhesus TF339-340 insert at the orthologous position (before Q337) was expressed in CRFK cells and challenged with the indicated viruses. Error bars, SD. ns, not significant; $^{**}p < 0.01$; student's unpaired t test. TRIM5α expression levels were analyzed by immunoblot (IB) against the C-terminal HA tag. The drop in anti-HIV-1 potency may be explained by decreased expression or stability of human TRIM5α containing the rhesus TRIM5α insertion.

bioRxiv preprint doi: https://doi.org/10.1101/2024.05.07.592993; this version posted May 10, 2024. The copyright holder for this preprint (which was not certified by peer review) is the author/funder, who has granted bioRxiv a license to display the preprint in perpetuity. It is made available under aCC-BY-NC 4.0 International license.

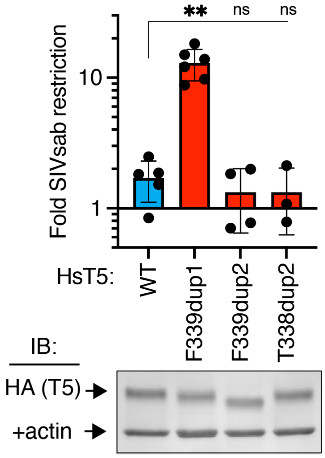

Figure S6: The functional effects of duplicating F339 are context-specific. Duplication of an additional residue before or after F339 (yielding amino acid sequence FVFV or TFTF) does not allow human TRIM5α to inhibit SIVsab when expressed in CRFK cells. Antiviral function does not correlate with TRIM5α expression levels, as analyzed by immunoblot against the C-terminal HA epitope tag. Error bars, SD. ns, not significant; $^{**}p < 0.01$; Welch ANOVA with Dunnett's T3 multiple comparisons test.

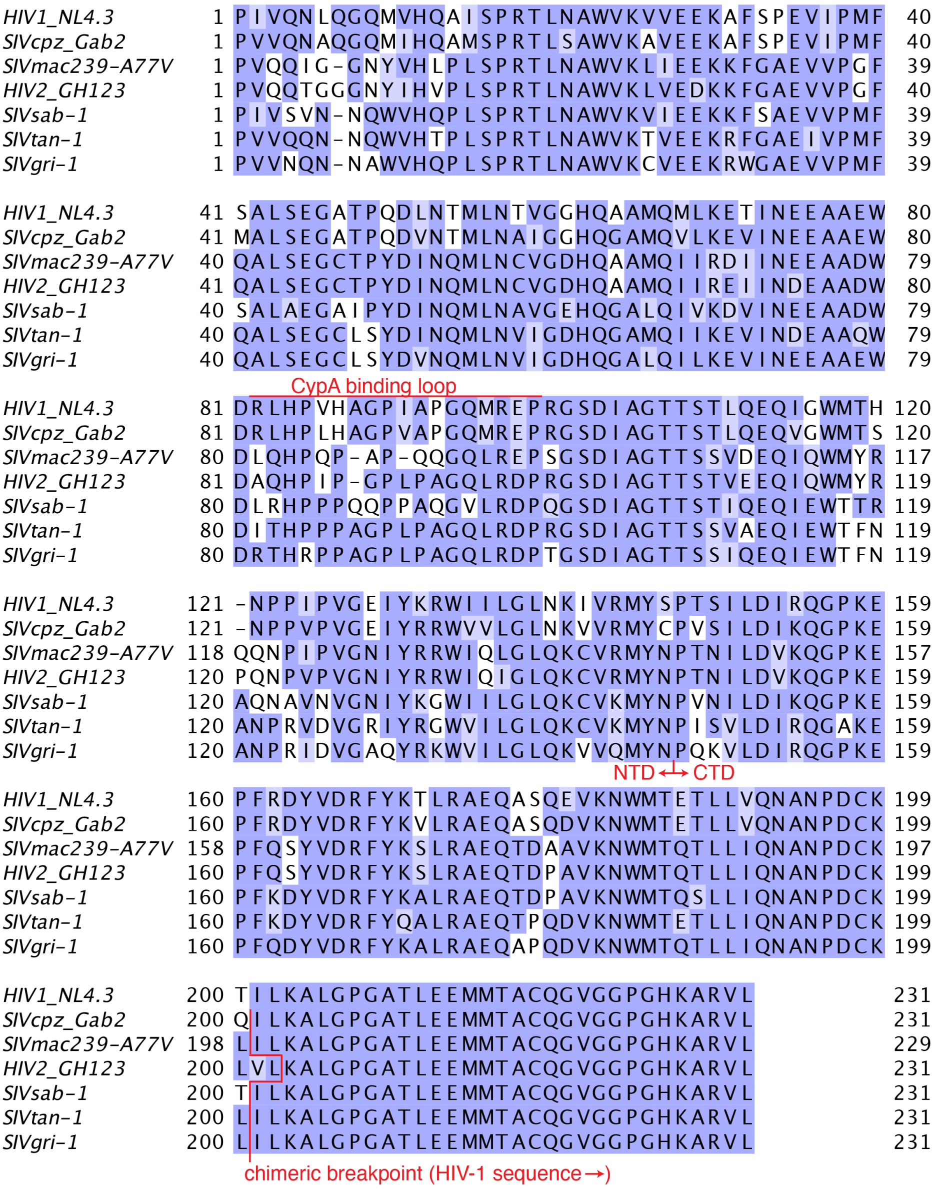

Figure S1: Lentiviral capsid sequences used in this study. All capsids were chimerized with C-terminal HIV-1 sequence at the indicated breakpoint and were expressed within the HIV-1 (NL4-3 strain) gag/pol to generate virus. The cyclophilin A (CypA)-binding loop, which has been implicated in TRIM5α recognition (*41*, *42*), and the domain boundary between the N-terminal domain (NTD) and C-terminal domain (CTD) are indicated. Residue conservation is colored by BLOSUM62 score.
